# FN1 governs global protein lactylation in acute myocardial infarction: a metabolic–epigenetic axis driving cardiac injury and impeding stem cell repair

**DOI:** 10.3389/fcell.2026.1897810

**Published:** 2026-07-30

**Authors:** Jia Ren, Linai Han, Jingjing Li, Zehui Wang, Haoyue Wang, Gangfei Han, Qinghua Han

**Affiliations:** 1 The First Clinical Medical College, Shanxi Medical University, Taiyuan, Shanxi, China; 2 Department of Cardiology, The First Hospital of Shanxi Medical University, Taiyuan, Shanxi, China; 3 Department of Cardiology, The People’s Hospital of Taiyuan, Taiyuan, Shanxi, China; 4 Key Laboratory of Cellular Physiology at Shanxi Medical University, Ministry of Education, Taiyuan, Shanxi, China; 5 Shanxi Innovation Center for Integrated Management of Hypertension, Hyperlipidemia and Hyperglycemia Correlated with Cardiovascular and Cerebrovascular Diseases, Taiyuan, Shanxi, China

**Keywords:** acute myocardial infarction, diagnostic biomarker, FN1, lactylation, mesenchymal stem cells, metabolic-epigenetic regulation

## Abstract

**Background:**

Acute myocardial infarction (AMI) triggers metabolic reprogramming, resulting in substantial lactate accumulation. This metabolite drives protein lactylation, a recently recognized post-translational modification that modulates cellular functions. However, the regulatory framework and primary drivers of lactylation in AMI, as well as their impact on myocardial repair, remain poorly defined. The hostile post-infarction microenvironment further limits the therapeutic efficacy of regenerative cell therapies, such as human endometrial mesenchymal stem cells (hEnMSCs). This study aimed to identify a central regulator of protein lactylation during AMI and develop a strategy for microenvironmental reprogramming to enhance stem cell-based interventions.

**Methods:**

Transcriptomic profiles from AMI patients were integrated with machine learning algorithms and single-cell RNA sequencing to identify core genes. Biological significance was validated using oxygen-glucose deprivation (OGD) cellular models and murine infarction paradigms, employing genetic silencing and lactate administration. The cooperative benefit of modulating the identified target in conjunction with hEnMSC delivery was subsequently assessed.

**Results:**

Our findings establish fibronectin 1 (FN1) as a functional bridge linking metabolic stress to epigenetic modification in AMI. Specifically, we discover that FN1 acts as a critical upstream regulator of global protein lactylation, a mechanism not previously recognized. Simultaneously, we acknowledge that FN1’s well-characterized roles in fibrosis, extracellular matrix remodeling, and scar formation may operate as a parallel, independent mechanism contributing to cardiac pathology. By showing that FN1 reduction specifically attenuates lactylation and that lactate supplementation reverses the protective effect, our data pinpoint lactylation as a key downstream effector of FN1’s detrimental function in AMI, while its classical functions likely remain active. Targeting this FN1-lactylation axis, particularly through combinatorial FN1 knockdown and stem cell therapy, offers a novel therapeutic strategy beyond conventional anti-fibrotic approaches.

**Conclusion:**

This work identifies FN1 as a critical upstream mediator of protein lactylation in AMI. Targeting FN1 not only directly attenuates tissue damage but also reshapes the local microenvironment to empower hEnMSC-mediated repair, offering a theoretical rationale for future preclinical studies combining FN1 targeting with stem cell therapy.

## 1 Introduction

Acute myocardial infarction (AMI) is a predominant contributor to cardiovascular-associated death and disability worldwide (Martin et al., 2025; Byrne et al., 2023; An et al., 2025). The fundamental pathological insult during AMI arises from sustained deprivation of oxygen and vital nutrients in myocardial tissue, leading to cellular necrosis following abrupt cessation of coronary blood flow (Martin et al., 2025; Byrne et al., 2023; An et al., 2025). Despite the rapid restoration of vessel patency via emergency percutaneous coronary intervention (PCI), subsequent pathophysiological processes—including reperfusion-triggered tissue injury, inflammatory cascade activation, and adverse ventricular remodeling—substantially increase the risk of heart failure, thereby drastically compromising long-term survival (Thygesen et al., 2018; Schulte and Mayrovitz, 2023; Hashmi et al., 2018). Notably, published evidence shows that the incidence of heart failure within 5 years after AMI ranges from 30% to 40% (Eser et al., 2022; Xing et al., 2024; Xuan et al., 2025). Therefore, transcending simple revascularization to achieve a comprehensive understanding of the intricate regulatory mechanisms underlying myocardial damage and repair, as well as to pinpoint novel therapeutic targets, represents a pressing clinical necessity for improving patient outcomes.

Tissue repair after AMI depends critically on the dynamic equilibrium of the local microenvironment within the injured zone. Recently, the role of metabolic network alterations in this process has drawn substantial attention. Under ischemic and hypoxic conditions, cardiomyocytes and infiltrating immune cells undergo a marked shift toward anaerobic glycolysis (the Warburg effect) to maintain energy production. This metabolic switch generates abundant lactate synthesis and accumulation, establishing a distinct biochemical milieu characterized by high lactate concentrations and reduced pH (Zhang et al., 2019; Wang et al., 2022; Zhang et al., 2023a). Historically regarded merely as metabolic waste and a contributor to intracellular acid load, lactate has undergone conceptual reevaluation based on emerging discoveries. It serves as a substrate, forming covalent bonds with lysine residues on proteins—a novel type of post-translational modification known as protein lactylation (Cheng et al., 2026; Su et al., 2025; Wang et al., 2025a). Functionally analogous to acetylation, this modification possesses broad capacity to regulate both histone and non-histone proteins, thereby influencing gene expression, metabolic pathway selection, and cell fate determination. Its status as a vital link connecting metabolic states to epigenetic regulatory mechanisms has been established within fields such as oncology and immunology (Wang et al., 2025a; Li et al., 2024; Chen et al., 2022). However, specific functions and regulatory mechanisms of lactylation in cardiovascular disease, particularly during AMI pathogenesis, remain largely unexplored.

Stem cell-based regenerative strategies offer potential for repairing damaged myocardium and attenuating adverse ventricular remodeling. Among diverse cell sources, human endometrial mesenchymal stem cells (hEnMSCs) exhibit considerable promise in early cardiac repair studies due to distinct advantages: abundant tissue availability, ease of harvesting, robust proliferative capacity, low immunogenicity, and minimal ethical concerns (Bausyte et al., 2023; Peng et al., 2024). Nevertheless, this therapeutic approach faces a pervasive and formidable obstacle: the extremely poor engraftment and survival of transplanted hEnMSCs within the hostile post-infarction microenvironment. Such severe, lactate-rich conditions expose MSCs to bioenergetic stress, trigger endoplasmic reticulum stress responses, and promote cell death, substantially curtailing therapeutic efficacy (Shokravi et al., 2022; Zhang et al., 2023b). Intriguingly, emerging evidence suggests that lactylation itself functions as a “dual-faced” regulator of MSC biology. Controlled modification may govern homing and engraftment properties, whereas excessive decoration can activate pro-death signaling cascades (Cheng et al., 2026; Wang et al., 2025a). This concept implies that “lactylation imprinting” may serve as a fundamental epigenetic switch connecting recipient metabolic adversity to stem cell functional fate. Yet, within the AMI context, the key molecular mediators orchestrating lactylation-dependent effects on hEnMSC function remain unidentified.

This study therefore aims to systematically dissect key regulatory nodes within the lactylation-dominated post-AMI microenvironment and explore whether targeted intervention can enhance and sensitize the therapeutic efficacy of hEnMSC engraftment. We began by integrating publicly available transcriptomic datasets, employing computational biology together with machine learning methodologies to screen for central factors simultaneously linked to lactylation dynamics and hEnMSC function. Next, we utilized single-cell analytical platforms to chart transcriptional profiles and temporal shifts of these critical genes with unprecedented resolution. Finally, through comprehensive *in vitro* and *in vivo* manipulations, we validated the role of fibronectin 1 (FN1)—the top candidate—in coordinating lactylation and cardiomyocyte injury, and assessed the synergistic therapeutic benefit achieved by combining FN1 knockdown with hEnMSC delivery following coronary artery occlusion. This investigation endeavors to reveal a novel regulatory axis of “metabolic stress—epigenetic modification—stem cell function,” thereby providing fresh conceptual frameworks and strategic approaches to overcome current limitations in cell-based therapy and develop combinatorial treatments for AMI.

## 2 Materials and methods

### 2.1 Data acquisition and preprocessing

The Gene Expression Omnibus (GEO; https://www.ncbi.nlm.nih.gov/geo/) is a publicly funded repository that archives functional genomics datasets, including those derived from microarray and high-throughput sequencing platforms (Cheng et al., 2023). Dataset selection adhered to the following predefined criteria: (i) expression profiles directly relevant to acute myocardial infarction (AMI); (ii) each dataset must contain both disease and healthy control specimens; (iii) samples must be exclusively of human origin. The training cohort was obtained under accession GSE66360 (file “GSE66360_series_matrix.txt.gz”), which comprises 49 AMI cases and 50 reference controls. An independent validation set was sourced from GSE106090 (file “GSE106090_RAW.tar”), consisting of 12 AMI samples and six baseline controls.

Raw intensity data were processed using the R package “affy” (version 1.74.0), which performed background adjustment and quantile normalization across all arrays. Platform-specific probe annotation files were retrieved from their respective sources. Probe identifiers were mapped to official gene symbols; probes lacking any corresponding annotation were excluded. When multiple probes mapped to the same gene, the arithmetic mean of their expression values was taken as the final expression level for that transcript. This procedure yielded a normalized gene expression matrix ready for subsequent analyses.

A manually curated set of 330 lactylation-associated genes was compiled from the prior publication by Cheng et al. (2023). Factors linked to human endometrial mesenchymal stem cells (hEnMSCs) were retrieved through the Gene Cards database (https://www.genecards.org/) (Stelzer et al., 2016), generating a collection of 31 core components closely related to hEnMSC biology.

### 2.2 Identification of differentially expressed genes (DEGs)

To compare transcriptional profiles between AMI and control samples within the training dataset, differential expression analysis was conducted using the R package “limma” (version 3.52.4) (Liu et al., 2021). For each transcript, adjusted P values (adj.*P*.Val) and log2 fold changes (log2FC) were computed. Differentially expressed genes were defined by thresholds that balance effect size and statistical reliability: adj.*P*.Val < 0.05 and |log2FC| ≥ 0.263 (equivalent to |fold change| ≥ 1.2) (Alivand et al., 2021).

### 2.3 Weighted gene co-expression network analysis to identify pathologically relevant modules

To pinpoint gene clusters whose expression patterns are tightly associated with AMI status, we performed Weighted Gene Co-Expression Network Analysis (WGCNA) using the training dataset. To ensure network robustness, the top 10,000 genes with the highest variability across samples—quantified by median absolute deviation (MAD)—were retained. After sample clustering and removal of outliers, an optimal soft-thresholding power (β) was selected so that the resulting adjacency matrix satisfied a scale-free topology criterion. Specifically, the soft-thresholding power was systematically evaluated over a candidate range powers = c (1:10, seq (12, 20, 2)); the optimal power β = 5 was selected as the lowest value achieving a scale-free topology fit index R^2^ > 0.85 while preserving high mean connectivity. Module detection was conducted via dynamic tree cutting (cutreeDynamic) with the following non-default parameters: deepSplit = 4, minClusterSize = 200 (i.e., minModuleSize = 200), and pamRespectsDendro = FALSE. The choice of deepSplit = 4 (more stringent than the default of 2) yielded moderately sized, biologically coherent modules amenable to downstream functional enrichment. Highly similar modules were subsequently merged using mergeCloseModules at a dissimilarity threshold of MEDissThres = 0.25 (corresponding to a correlation of 0.75 between module eigengenes). These parameter choices are consistent with WGCNA best-practice recommendations (Langfelder and Horvath, 2008) and were confirmed to produce stable, reproducible module assignments in our dataset. For each module, the relationship with AMI status (disease versus control) was evaluated by calculating the Pearson correlation between the module eigengene (ME) and the trait indicator. Modules that exhibited a significant correlation (|correlation coefficient| > 0.5) were retained, and all genes within them were extracted as a pool of “disease-associated genes” for downstream integrative analysis. A complete summary of all WGCNA parameters is provided in Supplementary Table S1.

### 2.4 Identification of lactylation-related genes with altered expression in the context of mesenchymal stem cell function

Overlap among the DEGs, WGCNA module members, and the lactylation-associated gene set produced a group of “AMI differential lactylation genes.” Next, Pearson co-expression analysis (*P* < 0.01) was performed between these differential lactylation genes and the hEnMSC functional gene set to identify AMI lactylation-linked genes that may influence mesenchymal stem cell activity.

### 2.5 Machine learning-based screening for diagnostic biomarkers

Three independent machine learning algorithms—LASSO (Least Absolute Shrinkage and Selection Operator), Random Forest (RF), and Boruta—were applied to further refine the list of candidate features for AMI classification. LASSO, a regularization method suited for high-dimensional data, was implemented with ten-fold cross-validation to select discriminative variables through penalty parameter tuning. Specifically, LASSO regression was performed using the R package glmnet (v4.1-7) with alpha = 1 and family = “binomial”. Ten-fold cross-validation (cv.glmnet, type.measure = “class”) was used to identify the optimal regularization parameter, with a fixed random seed (set.seed (123)) ensuring reproducibility. Both lambda.min (the λ yielding minimum cross-validated misclassification error) and lambda.1se (the largest λ within one standard error of the minimum) were computed. For our primary model, we adopted lambda.1se—in accordance with the “one-standard-error” rule—as it provides a more parsimonious model with enhanced generalizability by selecting fewer non-zero coefficients while maintaining predictive performance statistically indistinguishable from the minimum-error model. The coefficients at lambda.min are also reported in Supplementary Table S1 for completeness. Random Forest, an ensemble learning technique [ref], effectively identifies informative features while filtering out noise; genes with a relative importance score (MeanDecreaseGini) exceeding 2.5 (assessed via ten-fold cross-validation) were considered influential. The Boruta algorithm (Utkin and Konstantinov, 2022), which uses a random forest-based approach to determine the relevance of each feature, was also employed. LASSO and Random Forest analyses were carried out using the R packages glmnet (version 4.1-6), Boruta (version 1.7-1), and randomForest (version 4.7-1.1), respectively. The genes selected by all three methods were designated as core diagnostic markers for AMI.

### 2.6 Construction, validation, and interpretation of the diagnostic model

A logistic regression model was built using the expression levels of the core signature genes in the training set (GSE66360). The model was visualized as a diagnostic nomogram using the R package “rms” (version 6.3-0) (Robin et al., 2011). This nomogram translates individual gene expression values into points, and the total points are associated with the predicted probability of AMI, providing a practical tool for bedside risk estimation. Model discrimination was assessed via Receiver Operating Characteristic (ROC) curve analysis, with the area under the curve (AUC) calculated. AUC values between 0.7 and 0.9 indicate acceptable performance, while values greater than 0.9 reflect excellent accuracy [ref]. Internal validation was performed on the training dataset, and external validation was conducted on the independent GSE106090 cohort to evaluate generalizability. Calibration plots assessed the agreement between predicted probabilities and observed outcomes, and decision curve analysis (DCA) estimated the net clinical benefit across various threshold probabilities (Hu et al., 2024).

To interpret the impact of individual features on model predictions, the SHAP (SHapley Additive exPlanations) method was applied using the R package shapviz (version 0.9.1) [ref]. The XGBoost classifier was trained with the following hyperparameters: max_depth = 3, eta = 1, nrounds = 10, nthread = 2, and objective = “binary:logistic”, with a fixed random seed (set.seed (123)) for reproducibility. SHAP values were subsequently computed on the full training set using the shapviz package, which provides a unified, model-agnostic interface to TreeSHAP [ref] [ref]. Specifically, TreeSHAP—the exact, polynomial-time algorithm implemented within shapviz for tree-based models—was employed to decompose each prediction into additive feature contributions [ref]. SHAP-based feature importance was visualized using: (i) beeswarm summary plots (sv_importance with kind = “beeswarm”), (ii) bar plots of mean absolute SHAP values, (iii) SHAP dependence plots (sv_dependence) for individual feature effects, and (iv) waterfall and force plots (sv_waterfall, sv_force) for single-sample explanation. No additional approximation or sampling (e.g., Kernel SHAP background subsampling) was required, as TreeSHAP computes exact SHAP values for tree ensemble models. All SHAP parameters are listed in Supplementary Table S1.

### 2.7 Single-cell RNA sequencing analysis

To characterize myocardial cell composition and transcriptional patterns of key genes at single-cell resolution, we analyzed a previously published single-cell transcriptomic dataset derived from human cardiac tissue (Amrute et al., 2024). The raw count matrix was processed using the Seurat package (v5.0) for normalization, selection of highly variable features, and principal component analysis (PCA). Batch effect correction was performed using the Harmony algorithm to achieve data integration. Cell clusters were identified through unsupervised clustering and detection of well-established marker genes (MYH7, PECAM1, DCN), with stringent filtering applied to remove contaminating fibroblasts and macrophages. The cluster annotated as cardiomyocytes was reclustered and subsequently divided into six functional subsets based on literature: chamber-specific working cardiomyocytes, atrial-specific cardiomyocytes, conduction system cells, stressed/hypertrophic cardiomyocytes, lipid-metabolism-associated cardiomyocytes, and interferon-responsive cardiomyocytes. To investigate the dynamic transition of cardiomyocytes from a healthy to a diseased state, trajectory inference was performed using the Monocle 2 framework. After random downsampling of each subgroup, dimensionality reduction was performed with the DDRTree method to construct a cell state progression path. The region predominantly occupied by healthy ventricular cardiomyocytes was designated as the trajectory origin. Pseudotime values were assigned along this path, and the fluctuating expression intensities of the core signature genes were examined across the pseudotemporal continuum.

### 2.8 Cell maintenance and genetic perturbation

The AC16 human cardiomyocyte line (Procell) was propagated in DMEM/F12 medium containing 10% fetal bovine serum and 1% antibiotic-antimycotic, within a humidified incubator set to 37 °C and 5% CO_2_. To recapitulate ischemic injury, an oxygen-glucose deprivation (OGD) paradigm was implemented: standard medium was substituted with a glucose-depleted formulation, and cultures were placed in a tri-gas chamber (94% N_2_, 1% O_2_, 5% CO_2_) for 24 h. Sodium lactate was introduced at a 10 mM concentration concurrently with the onset of OGD (Zhang et al., 2025; Fan et al., 2023).

For functional interrogation of FN1, a short hairpin RNA sequence (5′-TGC​AGC​ACA​ACT​TCG​AAT​TAT-3′) was designed and synthesized. Lentiviral particles encoding this shRNA construct were generated and packaged by Hanbio Biotechnology Co., Ltd. AC16 cells were infected with these viral vectors and subsequently selected with puromycin (2.5 μg/mL) to establish stable FN1-knockdown (sh-FN1) and control (sh-NC) lines.

### 2.9 Immunoblotting

Cellular protein lysates were harvested in a lysis buffer supplemented with a 1% protease inhibitor cocktail. Protein concentrations were quantified using the bicinchoninic acid assay (Beyotime, #P0012S). Standard procedures for electrophoretic separation, membrane transfer, antibody hybridization, and chemiluminescent detection were followed. Primary antibodies used were: FN1 (CST, 26826), CDK6 (CST, 30483), Bax (CST, 2772), Bcl-2 (CST, 3498), and L-Lactyl Lysine (PTM Biolabs, PTM-1401). β-actin (CST, 4970) served as the loading control.

### 2.10 Evaluation of mitochondrial membrane potential (MMP)

Mitochondrial membrane potential was measured with a commercial assay kit (Beyotime, China). Following experimental treatments, cells were rinsed with PBS and incubated with JC-1 working solution at 37 °C for 20 min in the dark. After staining, cells were gently washed twice with the supplied buffer to remove excess dye and kept immersed in fresh buffer during image acquisition. Fluorescence images were captured under identical microscopic settings across all groups JC-1 aggregates (red fluorescence, indicating high MMP) and monomers (green fluorescence, indicating low MMP) were recorded.

### 2.11 Lactate dehydrogenase (LDH) release assay

Loss of plasma membrane integrity and consequent cellular necrosis were assessed by quantifying LDH efflux. Culture supernatants were collected and combined with the reaction mixture in a 96-well plate following the manufacturer’s protocol. After a 30-min incubation at room temperature in the dark, absorbance at 490 nm was recorded using a microplate reader. LDH release was expressed as a percentage relative to the maximum release obtained from lysed control samples.

### 2.12 Intracellular reactive oxygen species (ROS) quantification

Intracellular ROS levels were evaluated using the DCFH-DA fluorescent probe. Cell-permeable DCFH-DA is deacetylated by intracellular esterases to DCFH, which upon oxidation by ROS yields highly fluorescent DCF. After the respective treatments, cells were loaded with 10 μM DCFH-DA at 37 °C for 30 min in the dark. Following thorough PBS washes, fluorescence intensity was measured with excitation/emission wavelengths at 488/525 nm. Obtained values were normalized to the total protein content of corresponding wells.

### 2.13 Apoptosis assessment by flow cytometry

Cell apoptosis (late and early, corresponding to Annexin-V+ PI+ and Annexin-V+ PI− populations) was assessed by flow cytometry. Cells (1 × 10^6^ cells/well) were seeded overnight, centrifuged, and the pellet was resuspended. Subsequently, the cell suspension was stained with 5 μL Annexin V-FITC and PI (BD Biosciences) and incubated in the dark for 15 min. Apoptotic cells were detected using a flow cytometer (BD Biosciences), and data were analyzed using FACS software and FlowJo (version 10.6.2).

### 2.14 Isolation and culture of human endometrial mesenchymal stem cells

HEnMSCs were derived from human endometrial tissue (Peng et al., 2024). Briefly, digested tissue was processed to obtain a single-cell suspension, and density-gradient centrifugation was performed to enrich the mesenchymal stem cell fraction. This enriched population was plated in specialized medium and maintained under primary culture conditions at 37 °C with 5% CO_2_. The cells displayed a characteristic adherent, elongated, spindle-shaped morphology and robust proliferative capacity. Upon reaching 80 %–90% confluence, primary cells were dissociated and passaged at a 1:3 ratio after centrifugation at 1,300 rpm. Passages were numbered sequentially from P1 to P10. For cryopreservation, cells were trypsinized, resuspended in pre-cooled freezing medium, aliquoted, frozen in a controlled-rate freezer, and finally transferred to liquid nitrogen. For revival, cryovials were rapidly thawed in a 37 °C water bath, sterilized, transferred to complete medium, centrifuged, resuspended, and seeded into flasks for continued expansion at 37 °C under 5% CO_2_.

### 2.15 *In Vivo* experimental models

Eight-week-old male C57BL/6 mice were used in all experiments. Anesthesia was induced with inhaled isoflurane, and each animal was secured on a surgical platform. Endotracheal intubation was performed, and the mouse was connected to a rodent ventilator. A left thoracotomy through the fourth intercostal space exposed the heart, which was gently exteriorized. Permanent ligation of the left anterior descending (LAD) coronary artery was executed with a 7–0 silk suture placed approximately 2 mm below the left atrial appendage. Immediate blanching of the anterior left ventricular wall confirmed successful occlusion. The heart was carefully returned to the thoracic cavity, and the chest wall and skin were closed in layers. Sham-operated animals underwent identical procedures except for the ligation.

Experiment 1 (Functional validation of FN1 and lactate): Mice were randomly assigned to five groups (n = 5 per group): (1) sham; (2) MI model; (3) MI + shFN1, receiving tail-vein administration of shFN1-encoding lentiviral particles (1 × 10^9^ PFU/mL, 200 μL) 7 days before and 14 days after surgery; (4) MI + NaLac, receiving intraperitoneal sodium lactate (0.5 g/kg body weight) every other day post-surgery (Fan et al., 2023); (5) MI + shFN1 + NaLac, receiving both interventions.

Experiment 2 (Assessment of combined therapeutic strategy): Mice were randomly divided into three groups (n = 5 per group): (1) MI + sh-NC (control); (2) MI + hEnMSCs, receiving 1 × 10^6^ hEnMSCs (suspended in 50 μL PBS) via tail-vein injection on day 3 post-MI; (3) MI + shFN1 + hEnMSCs, with shFN1 lentiviral pretreatment before MSC transplantation.

At 28 days post-MI, cardiac function was assessed using a small-animal ultrasound system. Left ventricular end-diastolic diameter (LVEDD) and end-systolic diameter (LVESD) were measured, and left ventricular ejection fraction (LVEF) and fractional shortening (LVFS) were derived. Following echocardiography, animals were euthanized, and hearts were harvested. A portion of each heart was fixed in 4% paraformaldehyde, paraffin-embedded, sectioned, and stained with Hematoxylin and Eosin (H&E) and Masson’s trichrome to evaluate myocardial architecture and fibrotic collagen deposition. Remaining tissue was snap-frozen in liquid nitrogen for protein and RNA extraction. All animal procedures were approved by Wuhan Myhalic Biotechnology Co., Ltd. (approval number HLK-20251103-003).

### 2.16 Statistical analysis

All *in vitro* experiments were repeated independently at least three times. Data are expressed as the arithmetic mean ± standard error of the mean (SEM). For comparisons between two groups, an unpaired two-tailed Student’s t-test was used when data met normality and variance homogeneity assumptions; otherwise, the Mann-Whitney U test was applied. For comparisons among three or more groups, one-way analysis of variance (ANOVA) was performed. When variance homogeneity was confirmed, Tukey’s *post hoc* test was employed; if heterogeneity was detected, Welch’s ANOVA with Games–Howell *post hoc* comparisons was substituted. All computational bioinformatics analyses were conducted in the R environment. For gene expression levels or immune infiltration scores, the Wilcoxon rank-sum test was used for two-group comparisons, and the Kruskal–Wallis test for multiple-group comparisons. A P-value below 0.05 was considered statistically significant, denoted in figures as: *P < 0.05, **P < 0.01, ***P < 0.001.

## 3 Results

### 3.1 Transcriptomic analysis and WGCNA reveal altered gene patterns and key modules in AMI

**FIGURE 1:**
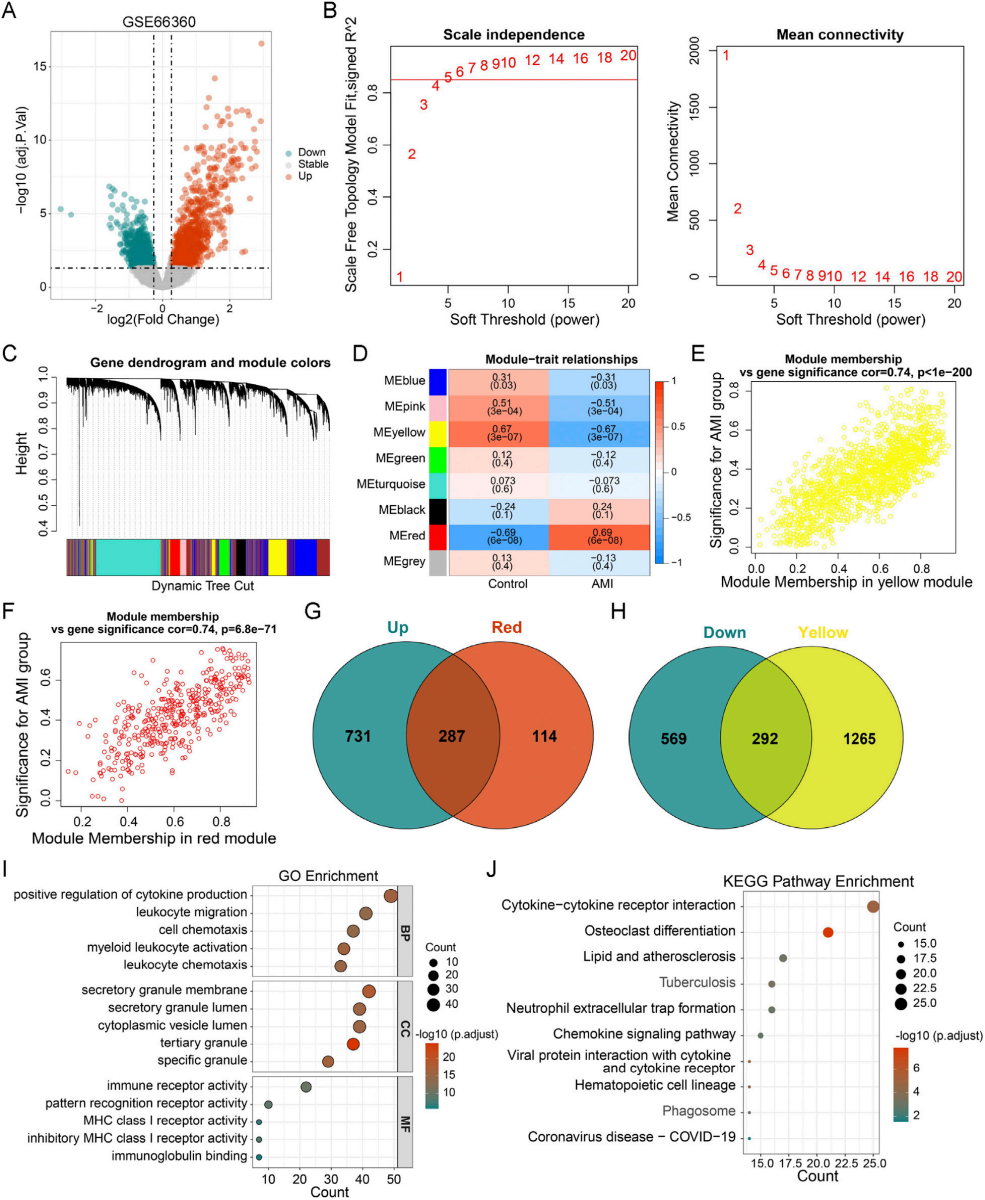
Differential gene expression and weighted gene co-expression network analysis in acute myocardial infarction. **(A)** Volcano plot showing differentially expressed genes between the acute myocardial infarction and control groups in the GSE66360 dataset. Upregulated genes are shown in red, and downregulated genes in dark green. **(B)** Left panel: Selection of the soft-thresholding power (β) for constructing the adjacency matrix. The x-axis represents the power value, and the y-axis indicates the squared correlation coefficient between log(k) and log(p(k)) for the network, where higher values indicate a better fit to scale-free topology. The red horizontal line marks the threshold of 0.85. Right panel: Mean connectivity across different soft-thresholding powers. The red line highlights the average connectivity at the selected power. **(C)** Gene clustering dendrogram showing co-expression modules identified by dynamic tree cutting, with distinct colors assigned to each module. **(D)** Heatmap displaying correlations between module eigengenes and the AMI phenotype (AMI vs. Control). **(E, F)** Scatter plots of module membership versus gene significance for the yellow **(E)** and red **(F)** modules. **(G, H)** Venn diagrams showing the overlap of red module genes with upregulated DEGs **(G)** and yellow module genes with downregulated DEGs **(H)**. **(I, J)** Bubble plots summarizing GO enrichment **(I)** and KEGG pathway enrichment **(J)** results for the 579 intersecting genes.

To comprehensively characterize the transcriptional changes accompanying AMI, differential expression analysis was first performed on the training dataset GSE66360. Applying thresholds of |log_2_FC| ≥ 0.263 and adjusted P < 0.05 identified 1879 differentially expressed genes (DEGs), consisting of 1018 upregulated and 861 downregulated transcripts (Figure 1A). Gene Ontology (GO) functional annotation showed that these DEGs were predominantly involved in biological processes such as “positive regulation of cytokine production,” “leukocyte migration,” “specific granule,” and “pattern recognition receptor activity” (Supplementary Figure S1A). Kyoto Encyclopedia of Genes and Genomes (KEGG) pathway mapping revealed significant enrichment in pathways tightly linked to inflammatory signaling, cellular stress responses, and cell fate determination, including cytokine-cytokine receptor interaction, the MAPK signaling cascade, and transcriptional dysregulation in cancer (Supplementary Figure S1B). Furthermore, using the CIBERSORT tool to deconvolute immune cell infiltration within tissue specimens, substantial disparities were observed in the relative proportions of diverse immune subsets when comparing AMI samples to controls. For instance, the fractional abundance of plasma cells and resting CD4 memory T cells exhibited marked shifts in the AMI setting (Supplementary Figures S2A–D), indicating extensive reprogramming of the immune surveillance network after myocardial infarction.

To identify additional gene clusters whose expression patterns are tightly coordinated with AMI status, WGCNA was performed. After evaluating various soft-thresholding powers, a value of 5 was chosen to construct a co-expression network satisfying scale-free topology requirements (Figure 1B). Based on this framework, all genes were subjected to dynamic tree cutting and partitioned into seven distinct modules, with the grey category representing unassigned components (Figure 1C). Assessing the correlation between each module’s eigengene and the AMI trait revealed that the red module (MEred) exhibited the strongest positive association with AMI, while the yellow module (MEyellow) displayed an inverse relationship (Figure 1D). These findings indicate that genes residing within the red module may serve facilitatory, disease-promoting roles during AMI pathogenesis. We subsequently examined two defining properties for red module constituents: Module Membership (MM, reflecting the concordance between a gene’s expression profile and the module’s overall pattern) and Gene Significance (GS, reflecting the concordance between a gene’s expression profile and the AMI trait). Results demonstrated a strong positive correlation linking MM with GS (r = 0.74, p < 1e^−200^) (Figures 1E, F), confirming the dense interconnectivity characteristic of the red module along with its pronounced relevance to AMI pathology. Intersecting red module members with all upregulated DEGs produced 287 genes, whereas intersecting yellow module members with all downregulated DEGs generated 292 genes (Figures 1G, H). Functional classification across this combined set of 579 genes showed marked enrichment in pathways such as positive regulation of cytokine production, IL-17 signaling cascades, the NF-κB relay pathway, and viral protein interactions with cytokines and their cognate receptors (Figure 1I,J), thereby strengthening the conclusion that inflammatory-immune cascades represent a core pathogenic axis in AMI.

### 3.2 Screening and identification of lactylation-associated hEnMSC functional core constituents in AMI

**FIGURE 2:**
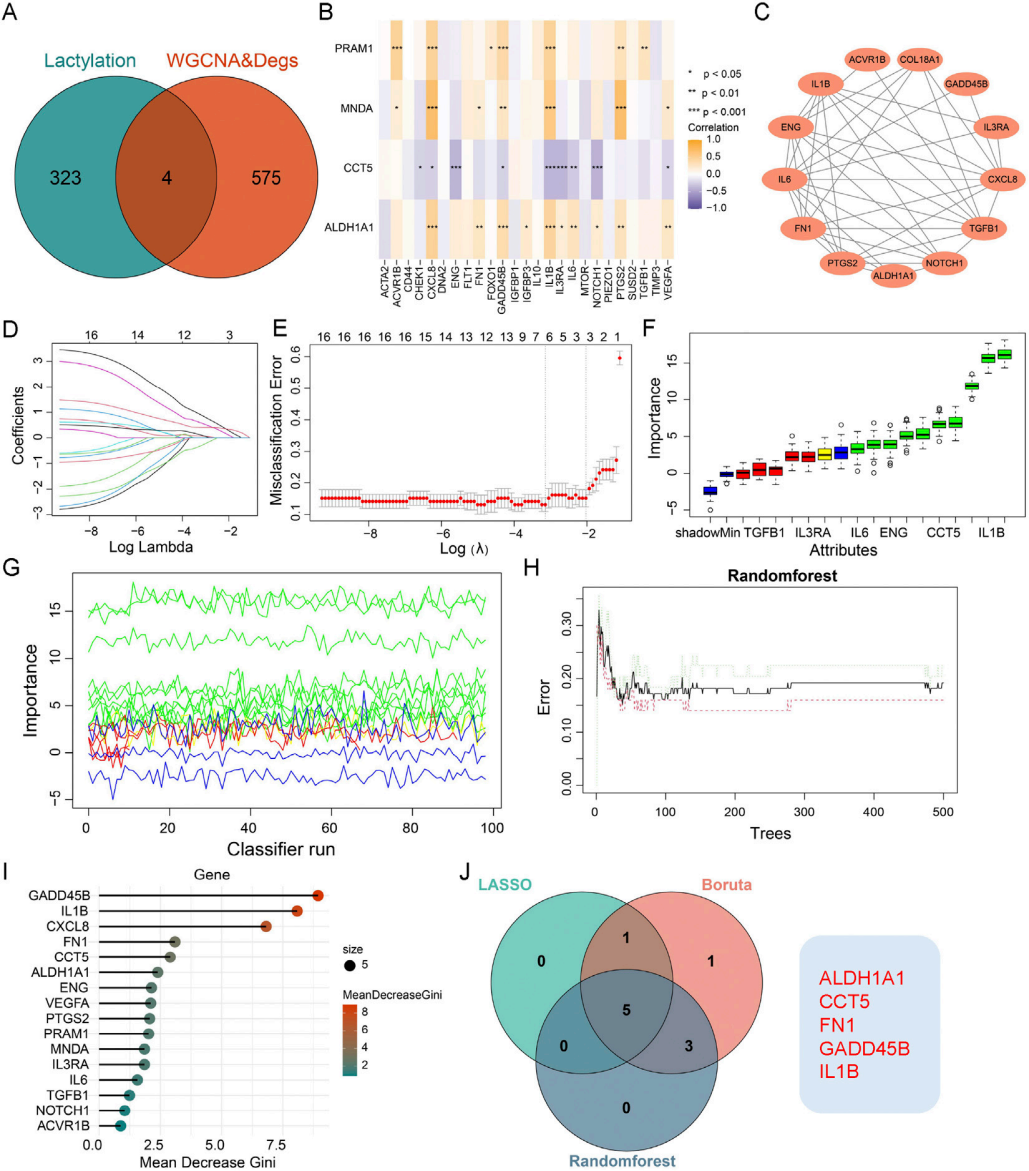
Screening and identification of lactylation-related functional core genes in hEnMSCs. **(A)** Venn diagram showing the overlap between AMI core-related genes and lactylation-related genes, identifying four differentially expressed lactylation genes. **(B)** Pearson correlation-based co-expression network (P < 0.01) between the four identified genes and hEnMSCs functional genes, revealing 16 core genes. **(C)** Protein-protein interaction network of the 16 core genes constructed using the STRING database. **(D)** Ten-fold cross-validation plot for tuning the regularization parameter (λ) in the LASSO model. **(E)** LASSO coefficient profile; the solid vertical line indicates the lambda value at the minimum partial likelihood deviance, and the dashed line marks the optimal lambda selected by cross-validation. **(F)** Final set of important features (genes) confirmed by the Boruta algorithm. **(G)** Plot of Z-scores representing the importance of all features during the Boruta feature selection process. **(H)** Plot of the out-of-bag (OOB) error rate against the number of trees in the Random Forest model. **(I)** Variable importance plot ranked by the mean decrease in the Gini index. **(J)** Venn diagram illustrating the intersection of results from the LASSO, Boruta, and Random Forest algorithms, ultimately identifying five core signature genes: ALDH1A1, CCT5, FN1, GADD45B, and IL1B.

We next focused on isolating key intersections linking lactylation events with hEnMSC functionality. First, the overlap between the previously identified 579 AMI-centric genes and the documented catalog of 330 lactylation-associated components was computed, revealing four elements that represent differentially expressed lactylation markers in AMI (Figure 2A). To establish connections between these markers and cardiac repair processes, Pearson correlation coefficients (P < 0.01) were calculated comparing them against 31 established hEnMSC functional genes. This analysis highlighted 16 constituents that simultaneously correlated with lactylation-related components and exhibited association with hEnMSC activity; these were subsequently termed AMI lactylation-linked hEnMSC functional genes (Figure 2B). A protein-protein interaction network constructed via the STRING database demonstrated extensive connectivity among these 16 entries (minimum confidence threshold 0.15), forming a densely interwoven nexus (Figure 2C), suggesting coordinated participation within shared biological programs.

To distill a parsimonious subset of genes possessing superior diagnostic discriminatory power from the 16 candidates, three complementary machine learning approaches were implemented. LASSO regression, employing tenfold cross-validation for variable selection, ultimately nominated six candidates (Figures 2D, E). The Boruta algorithm confirmed ten salient attributes relevant to AMI distinction (Figures 2F, G). The Random Forest method, relying on Gini coefficient importance ranking, identified eight top-ranking features (Mean Decrease Gini >2.5) (Figures 2H, I). Intersecting the outputs generated across all three methodologies yielded final validation of five quintessential signature genes: aldehyde dehydrogenase 1A1 (ALDH1A1), chaperonin containing TCP1 subunit 5 (CCT5), FN1, growth arrest and DNA damage inducible beta (GADD45B), and interleukin 1 beta (IL-1β) (Figure 2J). These five genes assumed priority status for subsequent functional validation and model construction.

### 3.3 Construction and validation of an AMI detection scheme based on core constituents

**FIGURE 3:**
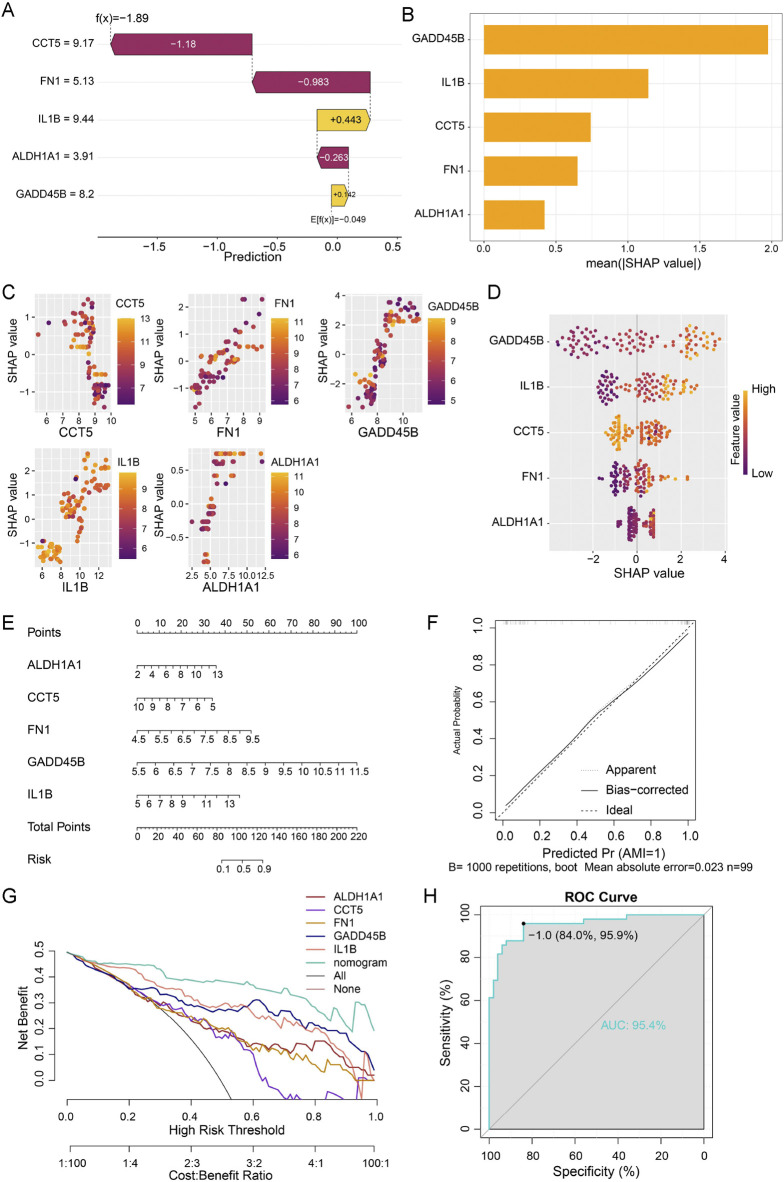
Construction and validation of an acute myocardial infarction diagnostic model based on core signature genes. **(A)** SHAP force plot for a representative sample, illustrating the direction and magnitude of each feature gene’s contribution to the individual prediction. **(B)** Bar plot showing the global mean absolute SHAP value for each feature gene, representing its overall contribution to the model. **(C)** SHAP summary (bee swarm) plot displaying the distribution of SHAP values (contribution to predicted risk) for each feature gene across all samples, colored by gene expression level. **(D)** SHAP dependence plot for IL1B, depicting the non-linear relationship between its expression level and the corresponding SHAP value. **(E)** Diagnostic nomogram for AMI based on the expression levels of the five core genes. **(F)** Calibration curve of the nomogram in the training set (GSE66360), evaluating the agreement between predicted and observed probabilities. **(G)** Decision curve analysis assessing the clinical net benefit of the model across a range of threshold probabilities. **(H)** Receiver Operating Characteristic (ROC) curve for internal validation within the training set.

Applying SHAP methodology to interpret the final predictive model, observations revealed that all five signature genes contributed to the forecasting output. When assessed individually, IL1B and GADD45B exerted positive influence, whereas ALDH1A1, CCT5, and FN1 imparted opposing effects (Figure 3A). However, upon integration into a holistic prediction, all five central constituents delivered favorable contributions toward the aggregate forecast (Figure 3B). The SHAP summary plot (swarm format) confirmed that the majority of the five signature genes populated the side representing positive SHAP values, indicating a positive contribution to model prediction (Figure 3C). Interaction plots across multiple factors demonstrated that the incremental forecasting impact mirrored the rising abundance levels of individual signature genes (Figure 3D).

To validate the combined diagnostic performance of the five signature genes for AMI detection, they were incorporated into a probability nomogram. Decision curve evaluation together with calibration plots indicated satisfactory accuracy for this nomogram (Figures 3E–G). ROC curve analysis demonstrated that the nomogram model achieved AUC values exceeding 0.7 in the training dataset, thereby affirming the integrated diagnostic and predictive utility of individual signature genes for AMI identification (Figure 3H). Additional immune analytics further revealed that, with the exception of CCT5, the remaining four signature constituents displayed significant positive associations with activated mast cell populations (Supplementary Figures S2C, D).

### 3.4 Functional associations of core constituents and identification of molecular subtypes among AMI patients

To gain deeper insight into the plausible biological involvements characterizing each member of the five-gene core signature set, Gene Set Enrichment Analysis (GSEA) was applied. Findings demonstrated that each constituent connected with discrete signaling pathways (Supplementary Figure S3). For instance, FN1 displayed prominent enrichment within “ECM-receptor interaction” and “Ribosome” pathways, harmonizing with its dual capacities as an extracellular scaffold element furnishing architectural reinforcement while possibly modulating the translational apparatus. IL1B displayed principal enrichment in Toll-like receptor signaling and Leishmaniasis infection pathways, underscoring its identity as a cardinal pro-inflammatory signaling molecule.

**FIGURE 4:**
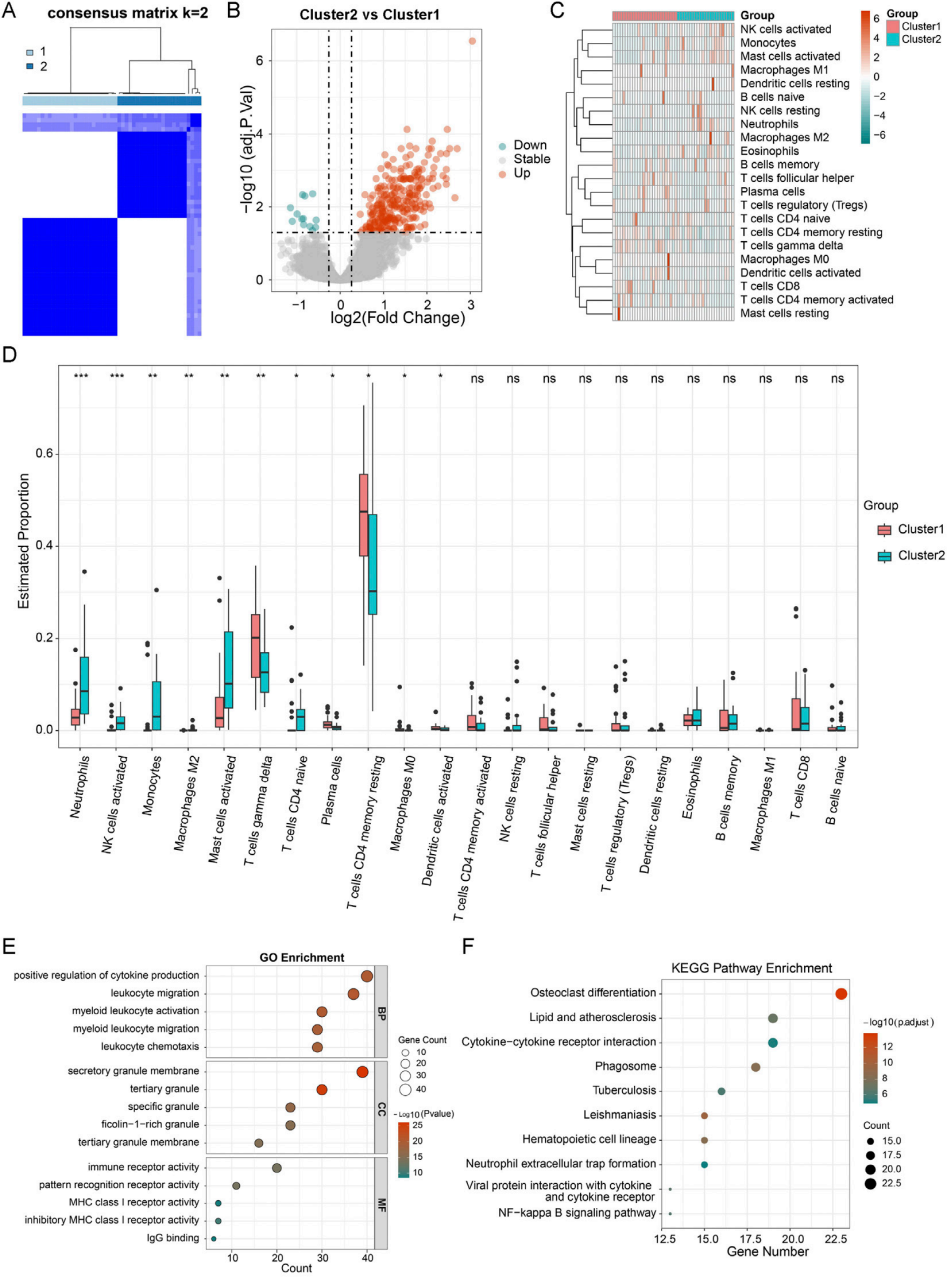
Identification of molecular subtypes in acute myocardial infarction based on core signature genes. **(A)** Unsupervised consensus clustering based on the expression of the five core genes identifies two stable molecular subtypes (Cluster 1 and Cluster 2). **(B)** Volcano plot of differentially expressed genes (DEGs) between the two subtypes. **(C)** Stacked bar chart showing the proportions of 22 immune cell types in all samples. **(D)** Stacked bar chart showing immune cell infiltration proportions in the two subtypes. **(E, F)** Functional enrichment analysis of DEGs between subtypes: bar plot of significant GO terms **(E)** and bubble plot of enriched KEGG pathways **(F)**. ns *P* > 0.05, *P < 0.05, **P < 0.01, ***P < 0.001.

Based on the transcriptional signatures spanning these five core constituents, unsupervised consensus clustering was performed to stratify AMI subjects into two robust categories: Category 1 and Category 2 (Figure 4A). Pronounced divergences typified core constituent expression patterns contrasting these categories, whereby Category 2 manifested broadly augmented abundance levels (Figure 4B). Extended evaluation of the immune composition characterizing the two categories uncovered elevated infiltration of neutrophils, activated natural killer cells, monocytic series, and engaged mast cells within Category 2, while Category 1 demonstrated relative enrichment for γδ T lineage, resting CD4 memory T cells, and immunoglobulin secretors (Figures 4C, D). Functional clustering of DEGs distinguishing these categories indicated that these genes principally participated in processes such as leukocyte trafficking, myeloid leukocyte activation, and immune docking functionality (Figure 4E). KEGG pathway mapping accentuated hematopoietic cell lineage specification, Toll-like receptor signaling, and neutrophil extracellular trap formation (Figure 4F). These findings suggest that core constituents located along the lactylation-MSC axis possess the ability to segregate AMI patients into subsets harboring unique inflammatory-immune signatures. Category 2 presumably represents a variant distinguished by amplified immune reactivity and overt inflammation, plausibly linked to less favorable clinical trajectories.

### 3.5 Single-cell transcriptomics reveals selective FN1 enrichment in pathological stressed cardiomyocytes

**FIGURE 5:**
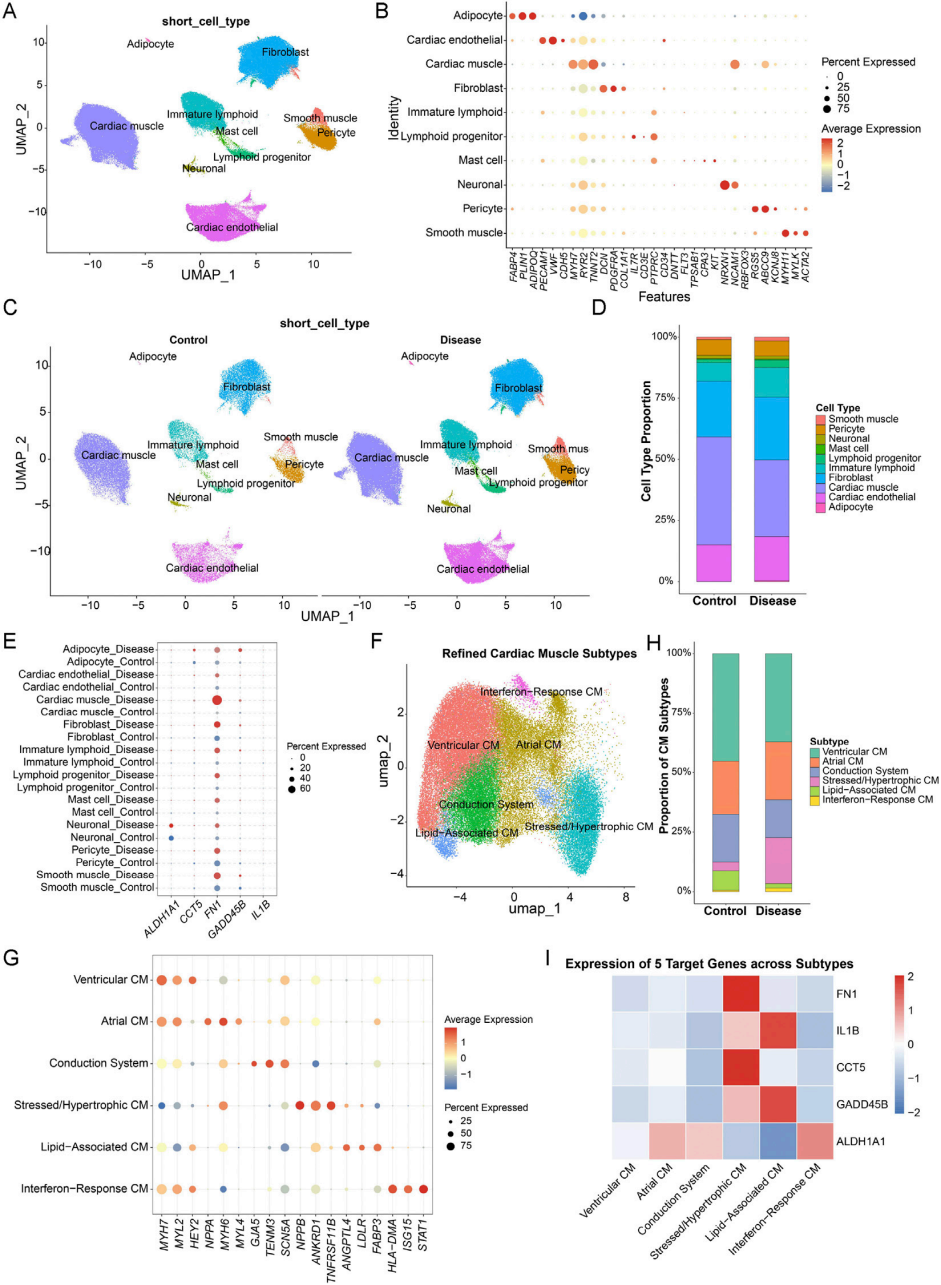
Single-cell transcriptomics reveals the post-myocardial infarction cardiac cell atlas and core gene expression patterns. **(A)** UMAP plot showing the clustering of all cells, annotated into 10 major cell types (e.g., cardiomyocytes, fibroblasts, endothelial cells) based on canonical marker expression. **(B)** Dot plot displaying the expression of classic marker genes for each identified cell type. **(C)** Split UMAP visualization comparing cell distribution between the control (left) and MI (right) groups. **(D)** Stacked bar chart illustrating the proportional changes of major cell types between MI and control conditions. **(E)** Dot plot showing the expression levels of the four candidate core genes (FN1, IL1B, ALDH1A1, GADD45B) across cell types and groups. **(F)** UMAP plot of re-clustered cardiomyocytes, revealing six distinct subsets. **(G)** Heatmap of characteristic marker gene expression for each cardiomyocyte subset. **(H)** Stacked bar chart comparing the proportions of cardiomyocyte subsets between MI and control groups. **(I)** Heatmap depicting the average expression of the four candidate genes across the six cardiomyocyte subsets.

To investigate microenvironmental shifts post-myocardial infarction (MI) at single-cell resolution, sequencing was performed on both MI and control cardiac specimens. UMAP dimensionality reduction coupled with clustering identified ten principal cell lineages, encompassing cardiomyocytes, vascular endothelial cells, and matrix-secreting fibroblasts (Figures 5A–C). Analysis of cellular composition demonstrated a pronounced increase in the fibroblast fraction within MI samples, indicative of active scar formation (Figure 5D). Examination of the five core signature genes revealed that FN1 was significantly upregulated in both cardiomyocyte and fibroblast populations derived from infarcted tissue (Figure 5E), suggesting a close association with a pathological state.

To precisely delineate FN1’s role within the cardiomyocyte compartment, reclustering analysis uncovered six discrete muscle subpopulations (Figure 5F). Beyond standard contractile ventricular cardiomyocytes, several disease-associated subsets were identified. The Stressed/Hypertrophic CM subpopulation, defined by high expression of NPPB and ANKRD1—established heart failure markers—exhibited selectively high expression of these markers (Figure 5G). This classification is supported by extensive literature: NPPB (encoding BNP) is a gold-standard clinical biomarker of myocardial wall stress and hypertrophy, with its expression strongly induced in failing hearts (Gertz, 2024); ANKRD1 (encoding CARP) is a mechanosensitive transcription cofactor rapidly upregulated in pathological hypertrophy and is widely used to distinguish stress-responsive cardiomyocytes (Lopes et al., 2024). The co-expression of these markers in a discrete subcluster therefore robustly identifies a clinically relevant, stress-activated cardiomyocyte state. Comparative assessment revealed a contraction in the proportion of normal Ventricular CMs alongside a marked expansion of the Stressed/Hypertrophic CM fraction in the MI cohort (Figure 5H), confirming a disease-driven shift toward a pathological phenotype. Crucially, transcriptional mapping unequivocally demonstrated that both FN1 and GADD45B were co-enriched within the Stressed/Hypertrophic CM subpopulation (Figure 5I), confirming a disease-driven shift toward a pathological phenotype. Crucially, transcriptional mapping unequivocally demonstrated that both FN1 and GADD45B were co-enriched within the Stressed/Hypertrophic CM subpopulation (Figure 5I). In contrast, IL1B and ALDH1A1 were primarily localized to other subsets linked to inflammation or metabolic adaptation. This positions aberrant FN1 expression squarely within the pathological cell state most strongly associated with cardiac pump dysfunction and ventricular wall thickening.

### 3.6 FN1 acts as a key driver of the pathological remodeling sequence in cardiac muscle cells

**FIGURE 6:**
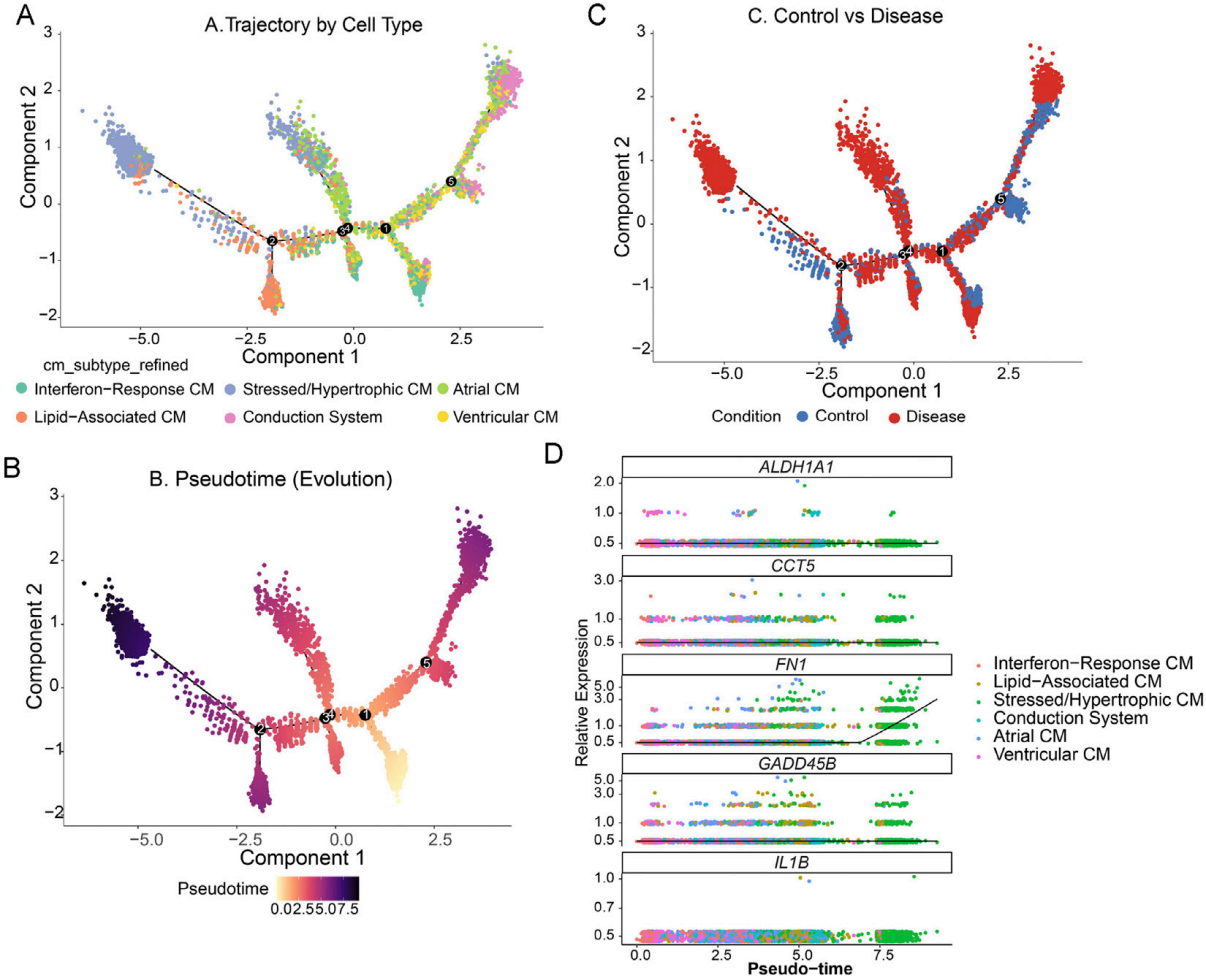
Dynamic evolution of cardiomyocytes after myocardial infarction and changes in core gene expression. **(A–C)** Pseudotime trajectory analysis based on Monocle 2. **(A)** Trajectory plot of cells in the reduced - dimensional space (DDRTree), colored by cell subset. **(B)** Trajectory plot colored by pseudotime. Dark blue represents early stages, while yellow represents late stages. **(C)** Trajectory plot colored by group. Blue represents Control, and red represents Disease. **(D)** Curve plot showing the dynamic expression of the core gene FN1 along the pseudotime trajectory, with the shaded area representing the confidence interval.

To delineate FN1’s variable contribution across advancing pathology, pseudotime trajectory reconstruction was performed using Monocle 2 (Figures 6A, B). The developmental continuum originated from healthy Ventricular CMs, predominantly populated by cells from control tissue, and culminated in a pathological state typified by Stressed/Hypertrophic CMs enriched within the MI cohort (Figure 6C). This graphically summarizes the transition of cardiomyocytes from a normal condition toward an aberrant terminal stage. Examination of dynamic transcriptional behavior revealed that FN1 abundance underwent a steady, pronounced increase concurrent with advancing pseudotemporal progression, reaching its maximum at the pathway’s endpoint (Figure 6D). This expression pattern indicates that FN1 activation does not represent an early event but rather constitutes a defining hallmark and plausible driving force behind the late-stage deterioration characteristic of maladaptive cardiomyocyte remodeling, providing kinetic evidence for its central role during disease escalation.

### 3.7 FN1 modulates global protein lactylation and mediates hypoxia-induced cardiac muscle damage

**FIGURE 7:**
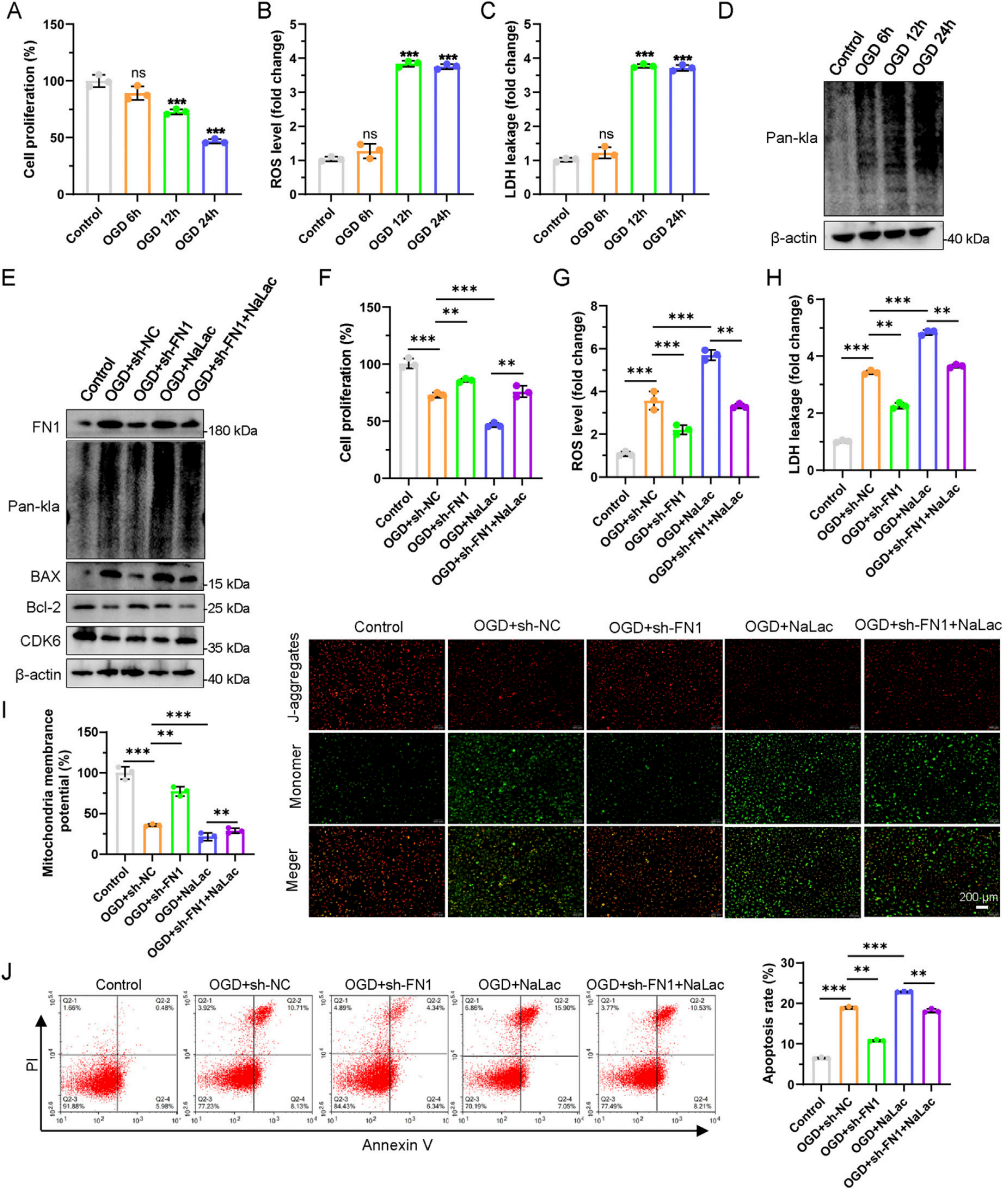
FN1 regulates lactylation and mediates hypoxia/reoxygenation injury in cardiomyocytes. **(A–D)** Time-dependent effects of oxygen-glucose deprivation (OGD) treatment on AC16 cells. **(A)** Cell viability (CCK-8 assay). **(B)** Intracellular reactive oxygen species (ROS) levels (DCFH-DA assay). **(C)** Lactate dehydrogenase (LDH) release rate. **(D)** Western blot detection of global protein lactylation levels. **(E)** Western blot detection of FN1, Bax, Bcl-2, and global lactylated protein levels in different treatment groups (Control, OGD, OGD + sh-NC, OGD + sh-FN1). **(F–J)** Rescue experiments involving FN1 knockdown and exogenous lactate treatment. **(F)** Cell viability. **(G)** Intracellular ROS levels. **(H)** LDH release rate. **(I)** Mitochondrial membrane potential (JC-1 assay, red/green fluorescence ratio). **(J)** Cell apoptosis rate (Annexin V/PI flow cytometry). Data are presented as mean ± SEM, n = 3. ns *P* > 0.05, **P < 0.01, ***P < 0.001 (One-way ANOVA with Tukey’s *post hoc* test).

Based on these findings, FN1 was selected as the prime candidate for functional validation. An oxygen-glucose deprivation (OGD) model in AC16 cardiomyocytes demonstrated that OGD exposure reduced cell viability in a time-dependent manner while simultaneously eliciting marked elevations in intracellular ROS, LDH release, and global protein lactylation signal intensity (Figures 7A–D). Immunoblot analysis confirmed that OGD treatment stimulated augmented FN1 protein expression (Figure 7E).

Decisive functional rescue experiments revealed that reducing FN1 abundance (shFN1) significantly counteracted OGD-induced reductions in cellular viability, ROS accumulation, LDH release, loss of mitochondrial membrane potential, and an increase in the apoptotic fraction (Figures 7F–J). The most consequential finding was that FN1 knockdown correspondingly curtailed the OGD-stimulated rise in global protein lactylation levels (Figure 7E). Conversely, exogenous supplementation with sodium lactate (NaLac) not only exacerbated OGD-inflicted damage and lactylation signal intensity independently but also completely abrogated the protective benefits conferred by FN1 depletion (Figures 7F–J). These results support the premise that FN1 functions downstream of lactate accumulation, acting as an essential node that modulates the extent of widespread lactylation and channels its detrimental effects toward cardiomyocytes.

### 3.8 Targeting FN1 ameliorates myocardial infarction injury and counteracts the adverse effects of high lactate in mice

**FIGURE 8:**
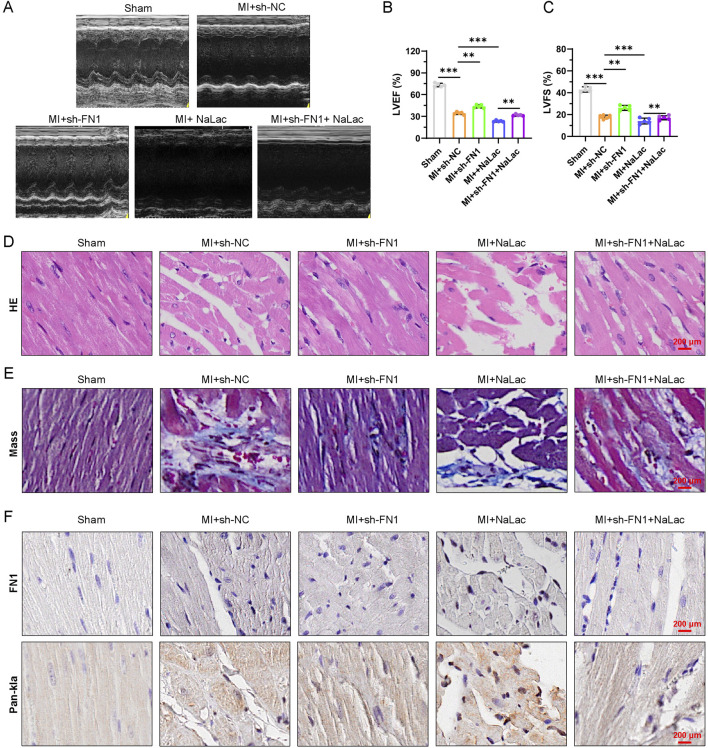
Targeting FN1 ameliorates myocardial infarction injury and counteracts the adverse effects of lactate in mice. **(A–C)** Assessment of cardiac function by echocardiography 28 days post-surgery. **(A)** Representative M-mode echocardiograms. **(B)** Statistical graph of left ventricular ejection fraction (LVEF). **(C)** Statistical graph of left ventricular fractional shortening (LVFS). **(D)** Representative image of H&E-stained myocardial tissue. Scale bars: 200 μm. **(E)** Representative image of Masson’s trichrome-stained tissue (collagen fibers in blue). Scale bars: 200 μm. **(F)** Representative immunohistochemistry images detecting FN1 and global protein lactylation (L-Lactyl Lysine) levels in myocardial tissue. Scale bars: 200 μm. Data are presented as mean ± SEM, n = 5. **P < 0.01, ***P < 0.001 (One-way ANOVA with Tukey’s *post hoc* test).

In a murine myocardial infarction (MI) model, suppressing FN1 significantly improved cardiac functional parameters (LVEF and LVFS) while reducing myocardial fibrotic deposition (Figures 8A–E). Corroborating the *in vitro* findings, FN1 knockdown correspondingly decreased global protein lactylation intensity detected in myocardial tissue samples (Figure 8F). Systemic administration of sodium lactate (NaLac) via the intraperitoneal route exacerbated functional decline in MI-affected rodents, whereas concomitant FN1 reduction effectively counteracted the detrimental consequences attributable to lactate treatment (Figures 8A–E). These observations robustly validate the existence of an “FN1-lactylation” functional axis within an intact organism: FN1 operates as a critical upstream regulatory element that amplifies the deleterious myocardial effects triggered by lactate.

### 3.9 Ablation of FN1 restructures the microenvironment and enhances the restorative efficacy of hEnMSCs

**FIGURE 9:**
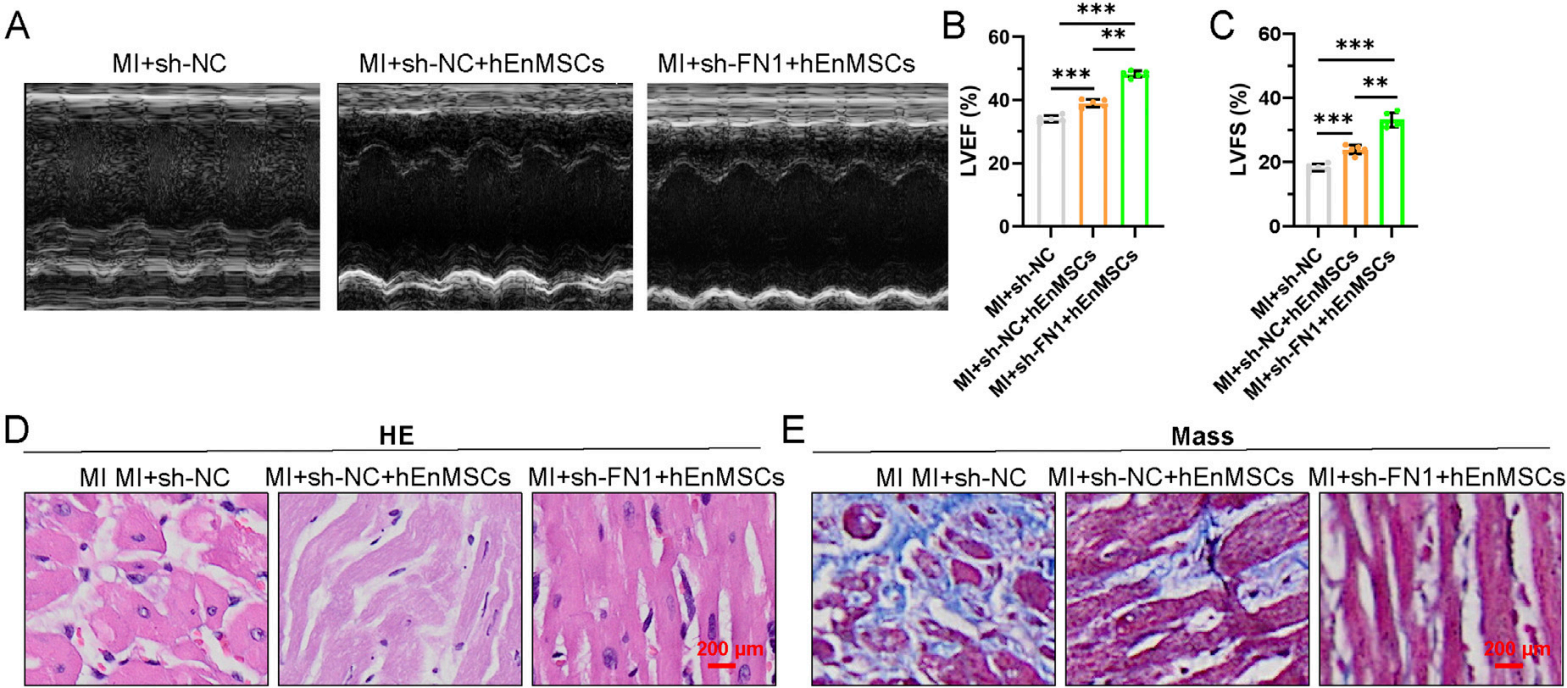
Knockdown of FN1 remodels the microenvironment and sensitizes the therapeutic efficacy of hEnMSCs in myocardial infarction. **(A–C)** Assessment of cardiac function by echocardiography 28 days post-surgery. **(A)** Representative M-mode echocardiograms. **(B)** Statistical graph of left ventricular ejection fraction (LVEF). **(C)** Statistical graph of left ventricular fractional shortening (LVFS). **(D)** Representative image of H&E-stained myocardial tissue. Scale bars: 200 μm. **(E)** Representative image of Masson’s trichrome-stained tissue (collagen fibers in blue). Scale bars: 200 μm. Data are presented as mean ± SEM, n = 5. **P < 0.01, ***P < 0.001 (One-way ANOVA with Tukey’s *post hoc* test).

In a hEnMSC transplantation treatment trial, administering hEnMSCs alone conferred only limited improvement in post-MI cardiac function. However, hEnMSC grafting performed against a background of FN1 knockdown produced a pronounced synergistic protective effect. The degree of functional enhancement and reduction in myocardial scar area observed in this cohort (MI+sh-FN1+hEnMSCs) surpassed the corresponding values recorded in the hEnMSC monotherapy group (MI+hEnMSCs) to a statistically significant extent (Figures 9A–E). This outcome indicates that lowering FN1 abundance within the infarct zone microenvironment—potentially achieved by attenuating adverse lactylation—can remodel the local habitat, rendering it more receptive to the survival, engraftment, and reparative activities executed by MSCs. Consequently, this considerably boosts the potency inherent in cell-based therapeutic strategies.

### 3.10 Molecular docking identifies stable candidate compounds targeting core genes

To explore potential chemical starting points for future FN1-based therapeutic strategies, we performed a preliminary molecular docking analysis on the core signature genes using the DrugBank database. Specifically, the corresponding protein structures for ALDH1A1, CCT5, FN1, and IL1B were retrieved from the Protein Data Bank (PDB) under accession codes 4X4L, CCT5, 3CAL, and 6Y8I, respectively. Following docking simulations, the binding energies between each protein and its respective compound were computed: −3.9 kcal/mol (ALDH1A1), −5.2 kcal/mol (CCT5), −5.9 kcal/mol (FN1), and −6.6 kcal/mol (IL1B). All target proteins demonstrated spontaneous binding with their corresponding active components, as indicated by negative binding energy values. Notably, the interaction energies for CCT5, FN1, and IL1B were lower than −4.5 kcal/mol, suggesting stable binding conformations for these protein–compound complexes (Supplementary Figure S4). These findings collectively indicate that the identified target proteins can stably associate with their respective active ingredients, supporting the structural stability of the predicted interactions. The docking data provide a molecular basis for further experimental validation. As a preliminary computational exploration, these data do not constitute proof of biological activity but rather provide a computational basis for future experimental validation *in vitro* and *in vivo*.

## 4 Discussion

Through systematic multi-dimensional profiling, comprehensive functional validation *in vitro* and *in vivo*, and detailed single-cell transcriptomic dissection, this study progressively delineates an unexpected role and central mechanism for FN1 across the pathological continuum of AMI. Our findings not only establish FN1 as a key diagnostic indicator but also, in a novel capacity, position this molecule as a nexus connecting metabolic stress, epigenetic modifications, and cellular behavior.

Our investigation began with the integration of bioinformatic strategies and machine learning approaches to precisely extract five core constituents—FN1, IL1B, ALDH1A1, CCT5, and GADD45B—from the complex transcriptional landscape of AMI. The strength of this selection methodology lies in its comprehensive perspective, which does not rely solely on isolated expression differences but instead combines transcriptional coordination patterns (WGCNA modules), documented biological annotations (lactylation marks and MSC functionality), and diagnostic requirements. The ultimately identified components have received prior recognition in the relevant literature. For instance, IL1B, a cardinal pro-inflammatory mediator operating downstream of the NLRP3 inflammasome complex, is known to drive the inflammatory surge following AMI (Amrute et al., 2024; Alexanian et al., 2024). ALDH1A1 participates in aldehyde detoxification and retinoic acid biosynthesis, linking it to oxidative stress counteraction and cytoprotective responses (Rosa et al., 2025). GADD45B, a stress-responsive polypeptide implicated in cell cycle arrest and genomic maintenance, exerts notable influence in cardiac remodeling-associated conditions (Du et al., 2025). The diagnostic framework constructed using these constituents demonstrated commendable performance across two independent datasets. Such results provide a plausible molecular tool applicable to early AMI detection while simultaneously suggesting that these constituents themselves represent critical disease drivers, warranting thorough mechanistic investigation.

Through cellular functional investigations, we elucidated the capacity of FN1 to serve as a pivotal functional regulator of global protein lactylation levels. Under conditions of oxygen deprivation or myocardial infarction, reduction of FN1 substantially diminished the overall protein lactylation burden, whereas exogenous lactate supplementation abrogated the protective effects resulting from FN1 depletion. It is noteworthy that the 10 mM lactate concentration used in our OGD model is within the physiological range observed in ischemic cardiac tissue (5–20 mM), supporting the clinical relevance of our experimental conditions. This pattern strongly suggests that FN1 acts functionally upstream of lactylation, potentially by modulating the availability of lactate or by influencing the activity of the lactylation machinery. However, the precise molecular mechanism connecting FN1 to lactylation remains to be elucidated. It is possible that FN1 knockdown reduces lactylation indirectly by suppressing glycolysis and decreasing intracellular lactate levels, thereby reducing substrate availability for lactylation enzymes. Alternatively, FN1 may directly influence the expression or activity of lactylation “writers” (p300/CBP) through integrin-mediated signaling. We propose that FN1, possibly operating through the integrin-FAK signaling pathway, may exert reciprocal modulation upon the activity of intracellular lactate metabolic enzymes (LDHA), the capacity governing lactate transport, or alternatively, impact directly the equilibrium between “writer” enzymes typified by p300/CBP, thereby potentially controlling the magnitude of this epigenetic modification (Qin and Zhou, 2022; Wang et al., 2025b; Dušková et al., 2014). These specific mechanistic connections are not directly supported by experimental data in the present study and should be regarded as working hypotheses to be tested in future investigations. Systematic studies—including measurements of intracellular lactate levels, LDH activity, and targeted molecular interaction assays—are needed to distinguish between these possibilities.

These results support the premise that FN1 functions upstream of lactate accumulation, acting as an essential node that modulates the extent of widespread lactylation and channels its detrimental effects toward cardiomyocytes. However, we acknowledge a critical limitation of the present study: direct measurements of intracellular or extracellular lactate levels were not performed. Consequently, the possibility that FN1 knockdown reduces global lactylation indirectly—by suppressing glycolysis, lowering intracellular lactate concentrations, and thereby limiting substrate availability for lactylation writer enzymes—cannot be excluded. Future studies should incorporate quantitative lactate assays (e.g., enzymatic or LC-MS-based methods) and metabolic flux analyses using [U-^13^C]-glucose tracing to determine whether FN1 influences lactate production, export, or both. Such experiments will be essential to dissect the direct versus indirect contributions of FN1 to the lactylation machinery.

Furthermore, a key limitation of the present study is that we did not identify specific proteins or pathways that are lactylated in an FN1-dependent manner. Global lactylation changes observed after FN1 knockdown may reflect widespread alterations in cellular metabolism rather than a targeted, site-specific modification. Known lactylation substrates in other cell types include glycolytic enzymes (such as GAPDH, PKM2), mitochondrial complex I subunits, histones (H3K18la), and metabolic regulators (LDHA, PDHA1) (Li et al., 2025; Wang et al., 2025c). In the context of AMI, dysregulation of these proteins could contribute to metabolic inflexibility, mitochondrial dysfunction, and epigenetic reprogramming. Future studies employing quantitative lactyl-proteomics (using mass spectrometry with pan-lactyl-lysine enrichment) are urgently needed to identify FN1-dependent lactylation targets and to determine the biological consequences of these modifications. Such data will be essential to move beyond global correlations and to establish a mechanistic causality linking FN1 to specific lactylation-mediated pathways.

Single-cell sequencing investigations next yielded robust cellular substantiation for the mechanism described above, considerably enriching its pathological interpretation. The data revealed that FN1 upregulation was not distributed evenly among all cardiomyocytes but instead was concentrated selectively at high levels within the “stressed/hypertrophic cardiomyocyte” subset, which expanded markedly under disease conditions. However, it is important to note that FN1 expression was also elevated in other cell types (fibroblasts) after MI, consistent with the widespread increase observed in Figure 5E. Thus, the current observation is based on visual assessment of expression patterns within the cardiomyocyte sub-clusters, rather than a rigorous statistical comparison of FN1 expression across all cell types. Future quantitative analyses—comparing both the percentage of expressing cells and mean expression levels across clusters—are needed to determine whether FN1 exhibits genuine cell-type selective enrichment in stressed cardiomyocytes. Such localization nonetheless suggests that anomalous FN1 expression constitutes an inherent property of cardiomyocytes undergoing pathological phenotype switching, rather than merely representing a paracrine consequence of fibroblast activation occurring in the surrounding milieu. Pseudotime mapping further charted its dynamic contribution: FN1 transcript levels rose progressively as cardiomyocytes transitioned from a normal condition toward a pathological terminus, reaching maximal abundance at the trajectory endpoint. This finding demonstrates that FN1 represents a critical late-stage event during myocardial restructuring, wherein its function may involve “locking in” or “driving” cardiomyocytes toward and sustaining a deleterious, failure-linked state. Hence, therapeutic strategies aimed at FN1 go beyond intervening in a pro-fibrotic factor and may exert direct effects upon the core pathological cellular compartment responsible for deteriorating cardiac performance.

Ultimately, our findings offer a direct and innovative avenue for enhancing AMI treatment, especially for surmounting the limitations inherent in cell-based therapy. We discovered that the microenvironment shaped by elevated FN1 expression not only physically obstructs repair but also, through upregulation of lactylation, establishes a metabolic-epigenetic milieu that suppresses cellular survival and function. This mechanistic insight explains why hEnMSC administration alone yields restricted therapeutic benefit. The dual-pronged advantage conferred by FN1 knockdown—directly safeguarding cardiomyocytes while simultaneously remodeling the local habitat—appears to facilitate improved cardiac outcomes when combined with hEnMSC transplantation. However, we acknowledge a critical confounding factor in the *in vivo* experimental design: shFN1 lentivirus was administered 7 days prior to MI induction, whereas hEnMSCs were injected 3 days post-MI. This temporal mismatch means that the observed benefits in the combination group may be primarily attributable to the early protective effects of FN1 knockdown (reducing initial ischemic injury, diminishing early-stage lactylation burden), with transplanted hEnMSCs potentially contributing only secondarily. Consequently, the term “synergy” should be interpreted with caution, as this design does not allow definitive attribution of the therapeutic improvement to cooperative mechanisms between the two interventions. Future studies employing simultaneous or systematically varied treatment schedules, combined with direct cell-tracking methods, are needed to rigorously assess whether true synergy exists between FN1 targeting and MSC transplantation. These results provide a robust theoretical foundation for a novel combinatorial treatment paradigm of “targeting FN1 (through pharmacological or genetic intervention) coupled with MSC transplantation,” aiming to maximize the potential of cell-based interventions by fundamentally altering the host microenvironment, though further optimized experimental designs are required to validate the synergistic principle.

Nonetheless, the present study has several limitations that warrant attention in future work. First, regarding the depth of mechanistic elucidation, although we positioned FN1 as an upstream modulator governing global lactylation occurrence, the specific downstream molecular pathways remain insufficiently resolved. Determining whether FN1, transducing signals primarily via integrin receptors, alters lactate dehydrogenase A (LDHA) catalytic activity, monocarboxylate transporter (MCT) expression, or directly governs the subcellular localization and function of histone acetyltransferase proteins such as p300/CBP (which serve as lactylation “writers”) through FAK/PI3K/AKT or ERK cascade activation demands deeper exploration employing proteomic screens, metabolomic profiling, and a series of intermolecular interaction studies (Zhang et al., 2026). Second, the possibility that FN1 itself serves as a substrate undergoing reciprocal lactylation-directed modulation was not examined herein. Identifying lactylation attachment sites on the FN1 polypeptide using mass spectrometric analysis and scrutinizing the consequent functional consequences through targeted residue mutagenesis approaches are important directions for future research that could reveal potential “lactylation-FN1” self-reinforcing circuits, thereby enabling a more complete portrayal of this regulatory network. Third, concerning the choice of experimental platform, our single-cell sequencing and primary functional validations were grounded in murine systems. Although these provide a robust foundation conducive to mechanistic exploration, inherent differences distinguish murine versus human cardiac physiology, pathological progression, and immunological reactivity. Future corroboration examining the safety and sustained efficacy of FN1-directed interference together with combinatorial stem cell application in additional clinically relevant large animal models represents an essential step toward translational realization. Lastly, our extraction of patient-derived data relied exclusively upon retrospective transcriptomic repositories accessible through public archives. Additionally, while we validated the diagnostic model using an independent dataset, the sample size of this validation cohort (n = 18) is relatively small, which may lead to overestimation of diagnostic performance and limits the generalizability of our findings. The limited representation of diverse demographics, comorbidities, and disease severities within this cohort further constrains the model’s applicability to broader patient populations. Therefore, caution should be exercised when extrapolating these results to unselected clinical settings. Future validation in large-scale, multi-center, prospectively collected cohorts with stratified sampling (e.g., by age, sex, comorbidities, and disease stages) is essential to confirm the robustness and clinical utility of the diagnostic model. Assembling prospective clinical cohorts that enable measurement of circulating FN1 levels alongside correlation with lactylation surrogate indicators holds the capacity to more immediately validate the translational promise and bedside relevance inherent in our discoveries.

In summary, this study progressively demonstrates the multifaceted roles of FN1 in AMI: as a diagnostic biomarker, an upstream regulator of global lactylation, and a key identifier of pathological cardiomyocyte subsets. These discoveries not only deepen the understanding of metabolic-epigenetic mechanisms in AMI but also point towards a new synergistic therapeutic direction by targeting FN1 to achieve both myocardial protection and microenvironment remodeling, holding significant theoretical value and clinical translation prospects.

## 5 Conclusion

In summary, this study demonstrates that FN1 serves as a critical upstream mediator of protein lactylation, a previously unrecognized role that distinguishes it from its canonical fibrotic functions. The true novelty of our work resides in the identification of the FN1-lactylation link, which provides a metabolic-epigenetic framework for understanding AMI progression. We also emphasize that FN1’s established roles in fibrosis and cardiac remodeling likely continue to contribute to the disease process in parallel. These discoveries highlight that targeting FN1 in AMI may simultaneously disrupt both its lactylation-mediated epigenetic effects and its classical extracellular matrix functions, offering a multi-pronged therapeutic opportunity. The combinatorial strategy of FN1 knockdown with MSC transplantation further leverages this dual understanding, aiming to remodel both the metabolic/epigenetic and structural microenvironments.

## Data Availability

The original contributions presented in the study are included in the article/Supplementary Material, further inquiries can be directed to the corresponding author.
