# Supplementary material for: FN1 governs global protein lactylation in acute myocardial infarction: a metabolic–epigenetic axis driving cardiac injury and impeding stem cell repair
Source: Front Cell Dev Biol. 2026 Jul 30;14:1897810. doi: 10.3389/fcell.2026.1897810 (PMC13469012; doi:10.3389/fcell.2026.1897810)
Supplement: Supplementary file 1 [file Supplementaryfile1.docx]

Supplementary Material

# 1 Supplementary Figures

**Supplemental Figure 1. Functional enrichment analysis of differentially expressed genes.**

(A) Bar plot of GO enrichment analysis, showing the main enriched terms for DEGs in biological process, cellular component, and molecular function categories. (B) Bar plot of KEGG pathway enrichment analysis, showing significantly enriched signaling pathways for DEGs.

**Supplemental Figure 2. Analysis of the immune microenvironment in acute myocardial infarction.**

(A) Heatmap of immune cell infiltration between AMI and control groups, deconvoluted using the CIBERSORT algorithm. (B) Box plots showing differences in immune cell proportions between groups. (C) Stacked bar chart of the proportions of 22 types of immune cell infiltration. (D) Heatmap showing the correlation between the expression of core signature genes (ALDH1A1, FN1, GADD45B, IL1B) and immune cell infiltration. ns *P* > 0.05, **P* < 0.05, ** *P* < 0.01, *** *P* < 0.001.

**Supplemental Figure 3. Gene set enrichment analysis of core signature genes.**

(A-E) GSEA result plots for each of the 5 core signature genes (FN1, IL1B, ALDH1A1, CCT5, GADD45B), showing representative KEGG pathways significantly enriched in samples with high expression of each gene.

**Supplemental Figure 4. Docking results, protein ligand binding structure; small ligand structure; binding pattern from left to right.**

# Supplementary Table S1. Detailed parameters for WGCNA, LASSO, and SHAP analyses.

| Method | Parameter | Value |
| --- | --- | --- |
| WGCNA | Package version | WGCNA v1.72-1 |
|  | Genes retained (MAD filtering) | Top 10,000 |
|  | Candidate soft-thresholding powers | c(1:10, seq(12, 20, 2)) |
|  | Soft-thresholding power (β) | 5 |
|  | Scale-free topology R² threshold | > 0.85 |
|  | deepSplit | 4 |
|  | minClusterSize (minModuleSize) | 200 |
|  | pamRespectsDendro | FALSE |
|  | TOM type | unsigned |
|  | Clustering method | average linkage (hclust) |
|  | Module merging threshold (MEDissThres) | 0.25 (i.e., correlation ≥ 0.75) |
|  | Dissimilarity measure | 1 − TOM |
| LASSO | Package & version | glmnet v4.1-7 |
|  | α (alpha) | 1 |
|  | Family | binomial |
|  | Cross-validation | 10-fold |
|  | type.measure | class (misclassification error) |
|  | Lambda selection (primary model) | lambda.1se (one-standard-error rule) |
|  | Lambda selection (also reported) | lambda.min |
|  | Random seed | 123 |
| SHAP | SHAP package & version | shapviz v0.9.1 (TreeSHAP) |
|  | SHAP algorithm | TreeSHAP (exact, polynomial-time) |
|  | Base model | XGBoost classifier |
|  | XGBoost: max_depth | 3 |
|  | XGBoost: eta (learning rate) | 1 |
|  | XGBoost: nrounds | 10 |
|  | XGBoost: nthread | 2 |
|  | XGBoost: objective | binary:logistic |
|  | Data used for SHAP computation | Full training set |
|  | SHAP visualization types | Beeswarm, Bar, Dependence, Waterfall, Force |
|  | Random seed | 123 |

Note: Random seeds were fixed at 123 for all stochastic procedures to ensure full reproducibility.
